# Traumatic brain injury in infants and toddlers, 0–3 years old

**Published:** 2011-08-25

**Authors:** AV Ciurea, MR Gorgan, A Tascu, AM Sandu, RE Rizea

**Affiliations:** ‘Bagdasar Arseni’ Clinical Emergency Hospital Romania

**Keywords:** cephalhematoma, children, depressed (‘ping–pong’) skull fracture, diastatic skull fracture, extradural hematoma

## Abstract

Object: Children 0–3 years old present a completely different neurotraumatic pathology. The growing and the development processes in this age group imply specific anatomical and pathophysiological features of the skull, subarachnoid space, CSF flow, and brain.

Most common specific neurotraumatic entities in children 0–3 years old are cephalhematoma, subaponeurotic (subgaleal) hematoma, diastatic skull fracture, grow skull fracture, depressed (‘ping–pong’) skull fracture, and extradural hematoma.

Methods: We present our 10 years experience in neuropediatric traumatic brain injuries, between 1999 and 2009, in the First Department of Neurosurgery and Pediatric Intensive Care Unit. Including criteria were children, 0–3 years old, presenting only traumatic brain injury. We excluded patients with politrauma, who require a different management.

Results: We present the incidence of these specific head injuries, clinical and imagistic features, treatment, and outcome. We found 72 children with diastatic skull fracture, 61 cases with depressed (‘ping–pong’) skull fracture, 22 cases with grow skull fracture, 11 children harboring intrusive skull fracture, 58 cephalhematomas, 26 extradural hematomas, and 7 children with severe brain injury and major posttraumatic diffuse ischemia (‘black–brain’). Usually, infants and toddlers present with seizures, pallor, and rapid loss of consciousness. First choice examination, in all children was cerebral CT–scan, and for follow–up, we performed cerebral MRI. We emphasize on the importance of seizure prevention in this age group. Children presenting with extensive diffuse ischemia (‘black–brain’) had a poor outcome, death occurring in all 7 cases.

Conclusion: Children 0–3 years old, present with a total distinctive pathology than adults. Children with head injury must be addressed to a pediatric department of neurosurgery and pediatric intensive care unit. Prophylaxis pays the most important role in improving the outcome.

## Introduction

Traumatic brain injury (TBI) is the leading cause of death and disability in children. Statistical analyses shows that almost half of patients with a TBI each year in the United Kingdom are children under 16 years, and approximately one third of the patients with cranial trauma per year in the United States are children aged between 0 and 14 years old [1,2].

The most common causes of TBI in children: falls, child abuse, motor vehicle accidents sport accidents, assaults, and instrumental delivery. Regarding age distribution of TBI, there are two risk groups: the first group aged between 0 and 4 years old, and the second 15–19 years old. Boys seem to be affected twice the rate of girls [1].

Traumatic pathology during the first 3 years of life is completely different when compared with adults. Raimondi emphasized the differentials between children and adults' pathology: ‘children are not young adults’ [3].

Specific pediatric scales, adapted according to age, must be used to correctly grade the severity of TBI in children. The most common neurotrauma pediatric scales are: Pediatric Coma Scale/Children Coma Scale (PCS)[4], Children's Coma Score (CCS)[5], Trauma Infant Neurological Score (TINS)[6], and Glasgow Coma Scale (GCS) [7]. The outcome is graded by using neurotrauma pediatric outcome scales, such as: KOSCHI (King's Outcome Scale for Childhood Head Injury) score [8], Glasgow Outcome Scale (GOS)[9], and modified Rankin score [10].

## Material|methods

We analyzed in a retrospective manner all the consecutive cases with TBI, aged between 0 and 3 years old, admitted into the Department of Pediatric Neurosurgery from ‘Bagdasar–Arseni’ Clinical Hospital, in Bucharest, between 1st of January 1999 and 31st of December 2008 (10 years).

Inclusion criteria were age 0–3 years, TBI, no history of previous head injury, no multiple trauma and no birth trauma. Infants with birth trauma were excluded. 

## Results

312 consecutive cases of children 0–3 years old were admitted. Most children presented with minor head injuries, 283 cases (90.70%).

### Etiology

The most common causes of TBI in children 0–l3 years old were falls, in 173 cases (55.45%), falls from the same level 102 cases (32.70%), and falls from other level 71 cases (22.75%). Motor vehicle accidents were the second cause of TBI in infants and toddlers, in 74 cases (23.72%), pedestrians 56 cases (17.95%), and passengers 18 cases	 (5.77%). Other cases were accidental struck of the head in 39 cases (12.50%), assaults/child abuse in 26 cases (8.33%). There were no bicycle or sport related TBI.  

**Table 1:** Causes of TBI in children

Causes	No. cases	% cases
Falls	173	55.45%
Falls from the same level	102	32.70%
Falls from other level	71	22.75%
Motor vehicle accidents	74	23.72%
Pedestrians	56	17.95%
Passengers	18	5.77%
Accidental struck of the head	39	12.50%
Assaults/child abuse	26	8.33%
Total	312	100%

**Figure 1:**
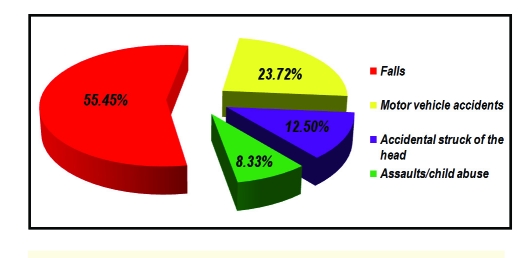
Causes of TBI in children.

### Traumatic brain injuries

There were 411 posttraumatic lesions in 312 children.

**Table 2:** Posttraumatic lesions in the study group

Injury	No. patients	% patients
Cephalhematoma	58	14.11%
Linear skull fracture	124	30.17%
Diastatic skull fracture	72	17.52%
Depressed skull fracture (ping–pong)	61	14.84%
Depressed skull fracture (cominutive)	19	4.62%
Grow skull fracture	22	5.35%
Penetrating head injury	11	2.68%
Extradural hematoma	26	6.33%
Subdural hematoma	–	–
Diffuse brain swelling	18	4.38%
TOTAL	411	100%

**Figure 2:**
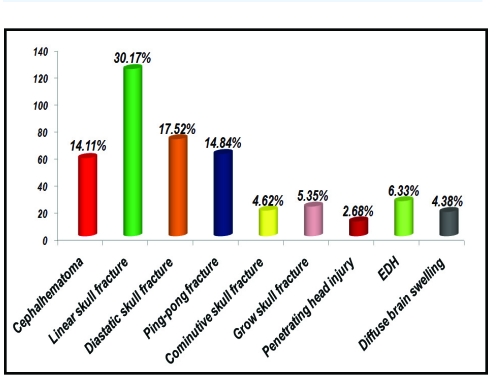
Posttraumatic lesions in the study group

### Cephalhematoma (CPH)

In our study group, we had 58 children (18.59%) with CPH

CPH, which does not spontaneously withdraw under conservatory treatment, requires surgical treatment. In persistent fluid, the hemorrhagic collection is evacuated by tapping (punctioning with a thick needle). In calcified CHP, surgery is performed for cosmetic reasons. A wide, arcuate skin incision in ‘horse shoe’, into the parietal area, on the border of CPH is performed. After a large scalp dissection, the calcified CHP is exposed. We perform a systematic resection of the calcified collection, with continuous wax hemostasis. For good cosmetic effects, level–adjustment of the parietal region is mandatory. No external drainage is needed.

### Skull fractures

Linear skull fractures were found in 124 children (39.74%). All children presenting in the emergency room with head trauma, and linear skull fracture on CT–scan or on skull X–ray, must be admitted in a Pediatric Neurosurgical Department because of the risk of developing extradural hematomas. Children with simple linear skull fractures without any other associated intracranial pathology did not require surgery. 

**Figure 3:**
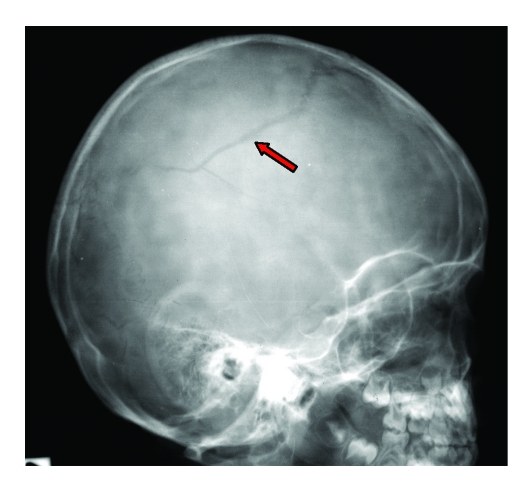
Linear right parietal skull fracture.

Diastatic skull fractures were found in 72 children (23.08%). In children aged between 0 and 3 years old, diastatic skull fractures carry a high risk of transforming into a growing skull fracture (GSF). All children with diastatic skull fracture were kept under careful observation for this reason. 

Depressed skull fractures (ping–pong) were found in 61 children (19.55%). In patients with ‘ping–pong’ fractures, a linear skin incision of 4 cm long, posterior to the depressed skull fracture and a burr hole in the middle of the incision, were made. By means of a periosteal elevator, introduced through the burr hole, into the epidural space beneath the fracture, the depressed bone was elevated.

Depressed skull fractures (cominutive) were found in 19 children (6.09%). Bone fragments were carefully elevated, and we performed duraplasty to cover dural lacerations. Brain laceration was resected. Bone fragments were fixed in place with wires. In some cases, bone fragments could not be replaced. We do recommend cranioplasty in children younger than 3 years old. 

Angiography with venous phase and CT–scan with coronal and sagittal reconstruction were done in all children with depressed fracture over the superior sagittal sinus (SSS), in order to evaluate the size, shape and patency of the sinus beneath the impacted bone. In depressed fractures across the SSS, a medial, rectangular free bone flap, centered over the SSS, is performed, the bone flap is carefully elevated and bone fragments are removed. The SSS is repaired by using Tachocomb to cover the rent within the sinus, a surgicel strip is packed over, and a periosteal graft is sewed over the sinus. Dural lacerations were sewed in narrow step or duraplasty is done, in order to perform a watertight dural closure. Bone flap was fixed with wires. External drainage can also be performed. Rapid repair of lesion of SSS is mandatory, because the risk of developing hemorrhagic shock is high in small children.

Grow skull fractures were found in 22 children (7.05%). 

## Case report

We report a case of a 5 weeks old girl, with a history of minor head trauma, 2 weeks before admission. The child presented with a progressive growing right frontal cystic, nontender mass, underlying palpable bony defect. CT–scan and 3D reconstruction CT–scan showed a diastatic skull fracture, leptomeningeal cyst, and brain herniation protruding through the bone defect.

**Figure 4:**
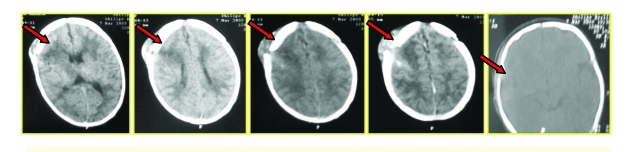
Diastatic skull fracture. Leptomeningeal herniation through diastatic fracture. Brain contusion

**Figure 5:**
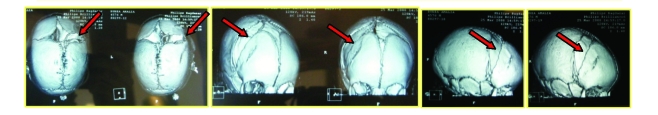
Disjunction of the coronal suture, reaching up to the anterior fontanelle

The patient's age, history of head trauma, progressive growing mass following trauma, clinical examination at admission and imagistic studies led to the diagnostic of GSF. Surgery was performed in 2 days time after admission, under general anesthesia. 

After the ‘horse shoe’ skin flap is centered over the fracture, the leptomeningeal cyst protruding through the bone defects rims was indentified. The cyst was punctioned, in order to evacuate CSF and to release pressure of the cystic mass. After the cyst's collapse, the borders of the skull fracture were revealed. The fracture was extended to a craniectomy, until free borders of the normal dura on both sides of the cranial defect were exposed. Lacerated dura and herniated brain laceration were resected. Duraplasty was performed with periosteal graft and the wound was closed in multiple layers. 

**Figure 6:**
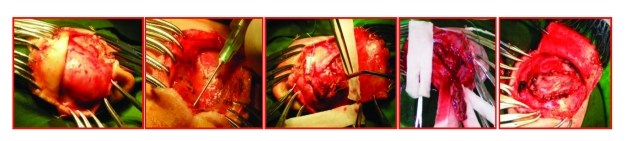
Leptomeningeal cyst protruding through the fracture borders, immediately under the skin. Punction of the cyst and CSF evacuation. Lacerated dura and lacerated brain removal. Duraplasty with periosteum.

The postoperative outcome was favorable, without neurological deficits or seizure under therapy. 

**Figure 7:**
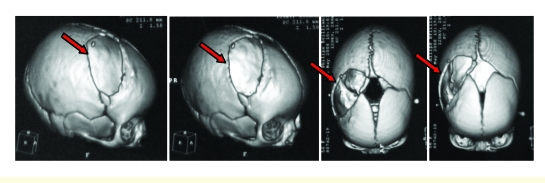
Postoperative 3D reconstruction CT–scan. Frontoparietal craniectomy.

### Craniocerebral wounds in children/Penetrating head injury

A total number of 11 children (3.53%) had craniocerebral wounds/penetrating head injury. Surgery in penetrating head injuries consisted of skin incision, beginning from the corners of the wound, with resection of contused borders of the wound, followed by the extraction of the superficial foreign bodies, and hemostasis. Bone fragments are elevated. Lacerated dura was removed; brain hematomas, clots, foreign bodies and cerebral decrypts were removed by lavage with saline and mild suction. Dura mater is closed tight and suspended. In large dural defects duraplasty, with periosteum patch, lyophilized dura, temporal fascia, fascia lata or artificial dura, was needed. For this, a sutured to the borders of the normal dura mater can be used. Bone fragments were repositioned, and fixed with wires.

### Extradural hematoma (EDH)

A number of 26 children (8.33%) had EDH

**Figure 8:**
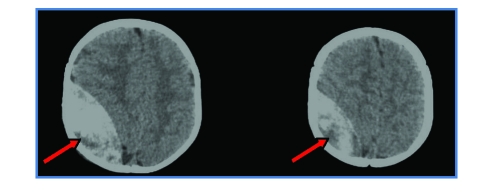
Right parietal extradural hematoma (preoperative imaging)

**Figure 9:**
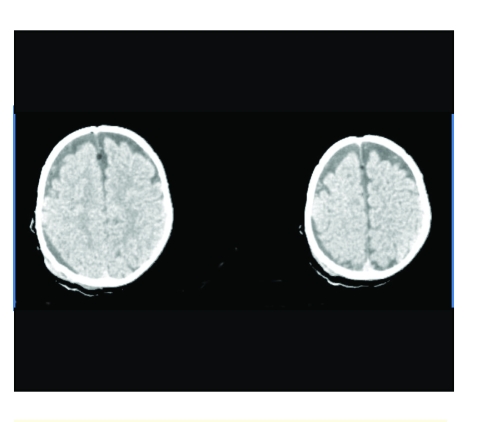
Right parietal extradural hematoma (postoperative aspect).

We had 3 cases with EDH presenting with hemorrhagic shock. 

### Clinic

**Table 3:** Clinical aspects in children with EDH.

Symptoms	No. patients	% patients
Pallor	26	100% (26/26)
Irritability /agitation/crying	15	57.69% (15/26)
Seizures	5	19.23% (5/26)
Somnolence / loss of consciousness	12	46.15% (12/26)
Vomiting	12	46.15% (12/26)
Fullness of fontanel	11	42.31% (11/26)

**Figure 10:**
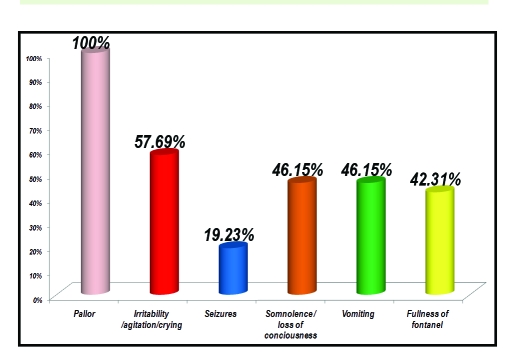
Clinical aspects in children with EDH

Classical surgery was used for convexity temporal EDH. A trauma flap was done (‘question mark’ skin incision: starting from the zygomatic arch, 1 cm anterior the tragus, in order to spare the facial nerve branch of the frontalis muscle and the anterior branch of the superficial temporal artery, curved posterior at the top of pinna, 4–5 cm behind the pinna, proceeded superiorly 1–2 cm ipsilateral to the midline, curved anteriorly and ended at the hairline, incision of the temporal muscle and frontotemporal craniotomy). EDH is evacuated by abundant lavage with saline and suction. One of the main goals of surgery is coagulation of the bleeding source. If the bleeding source is not found, it is assumed to be caused by the bleeding from the inner surface of the skull. Usually, secondary to reexpansion of the brain rebleeding is hindered, but application of bone wax to the inner surface of the skull or reflection of a free bone flap along the limbus of the hematoma may be performed. Dura mater was anchored and bone flap was repositioned and fixed.

Surgery for posterior fossa EDH consisted of vertical median or paramedian skin incision, suboccipital craniectomy, evacuation of the hematoma, hemostasis, and dura mater anchoring.

#### Diffuse brain swelling

18 children (5.77%) had diffuse brain swelling. In 7 children the CT–scan revealed extensive diffuse ischemia (‘black brain’). They were all comatose and had a poor outcome, death occurring in all 7 cases.

**Figure 11:**
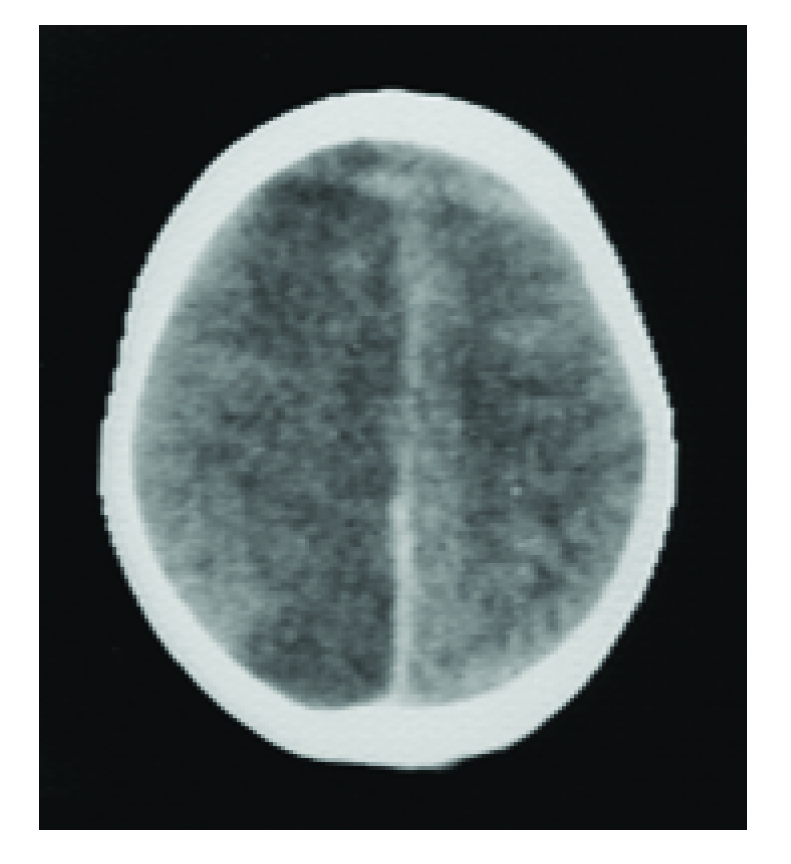
Diffuse brain swelling. CT–scan shows extensive diffuse ischemia (‘black brain’).

## Discussion

Infants and toddlers had some anatomical and functional particularities of central nervous system (CNS). Due to age–related particularities, a specific, distinctive posttraumatic response to external damaging factors occurs, completely different in children than in adults. 

Children's brain has a volume of 365 cm^3^ at birth, while adults have 1600 cm^3^. The weight in newborns is of 372 grams; in adults is of 1450–1500 grams. Newborns' brain has 100.000.000.000 (10^11^) neurons.

Infants and toddlers tolerate larger space–occupying traumatic lesions compared with adults, but consequences are similar.

Cephalhematoma (CPH) occurs in newborns and it is a subperiosteal hemorrhagic collection, caused by the rupture of blood vessels between skull and periosteum. Sometimes, an underlying linear skull fractures may also be found. Usually, CPH is found over the parietal bone and it is unilateral. CHP is limited to only one bone by periosteal attachment to the suture lines. CPH occurs within hours after delivery as a unilateral, soft, fluctuant swelling located in the parietal area. Small CPH requires no treatment. Most CPH spontaneously withdraw within the first three months. Persistent or infected collection requires evacuation through incision or tap. Tapping (punctioning with a thick needle) evacuates the hemorrhagic collection. Calcified CPH requires surgery. 

### Skull fractures

Depressed skull fracture may occur following difficult labor, but these patients were not included in our study. 

Linear skull fractures in children 0–3 years old are common, but usually have no clinical significance and require no specific treatment. Children with linear skull fracture must be admitted and followed up in a Department of Pediatric Neurosurgery for extradural hematoma occurrence. Diastatic skull fractures carry a high risk of transforming into a growing skull fracture (GSF) in this age group. 

Depressed skull fractures may be closed (simple fracture) or open (compound fracture). Depressed skull fractures, with or without focal neurological deficits or seizures, require surgical elevation, according to the depth of deformity. The ‘Ping–pong’ fracture is a particular type of depressed fracture found in newborns. It is usually encountered in parietal bone, and requires surgery. 

The depressed fracture, closed or compound, situated over the superior sagittal sinus (SSS) requires an angiography with venous phase or a CT–scan with coronal and sagittal reconstruction, in order to evaluate the size, shape and patency of the sinus beneath the impacted bone. Repairing of SSS must be performed rapidly, in order not to lose blood (in children there is a bigger risk of developing hemorrhagic shock).

A compound fracture, with depressed bony fragments and dural laceration needs emergency surgery. CT–scan can reveal associated hematomas, which require surgical evacuation. Brain laceration carries the risk of subsequent epilepsy, and must be resected. If bone fragments could not be replaced, cranioplasty with methylmethacrylate can be performed within 6 months after initial surgery. We recommend cranioplasty only in children older than 3 years. 

#### Growing skull fracture 

The grow skull fracture (GSF), is a rare complication of the skull fracture, mainly encountered during infancy and early childhood, in children under three years old [11–14]. The incidence of GCS is of 0.05 to 1.6% of all skull fractures during childhood [12,13,15,16]. 

The most common causes of GSF are head trauma secondary to falls, road traffic accidents and instrumental delivery [17–20]. Grow skull fractures are usually located at the cranial vault, into parietal or frontoparietal areas, but skull base [15,16,21,22] and posterior fossa [22,23,24] can also be involved.  

It is a progressive enlargement of diastatic fracture, along with leptomeningeal cyst and brain hernia. 

The period of time between head injury and GSF diagnosis can go up to many years [25,26]. Usually, GSF develop within 3–4 months following TBI. Positive diagnosis is made on history of head trauma, followed by progressive enlargement of skull fracture and leptomeningeal cyst protruding through the bone defect. 

Factors contributing to GSF occurrence are rapid brain growth and brain pulsation, found in young infants and children [27].

Pathogenesis consists of three phases. Within the first phase, following TBI, a linear skull fracture with periosteum tear and dural laceration occurs. Then, in time, fracture healing is hindered by intracranial hypertension and constant pulsation of the CSF, which favors invagination and entrapment of arachnoids into the diastatic fracture. During the third phase, patients present important bone diastasis, dural defect, progressive leptomeningeal and brain herniation through the diastatic fracture preventing apposition of bone, and elevated intracranial pressure [28]. In time, cerebroventricular changes occur, such as reactive gliosis into the underlying brain, with a subsequent development of porencephalic cavity [13,18,25,29]. 

During the first phase, patients have bone diastasis and seizures, the second phase presents with bone diastasis, seizures and motor deficits and the last phase with large bone diastasis, focal neurological signs and intracranial hyperpressure. 

Skull X–ray, cerebral CT–scan, with bone window and 3D reconstruction reveal the diastatic fracture and leptomeningeal and brain herniation.

Surgery is always required in patients with GCS to prevent seizures and neurological deficits occurrence. The key step is tight closure of dura mater. When dura mater is lacerated and cannot be sutured, the lacerated dura is resected, and duraplasty with periosteum, free flap or pediculated or artificial dura is performed. We recommend dural closure with free flap periosteum, sutured watertight to the normal dura mater, because it is an autologous tissue and it is the most convenient dural substitute. Watertight dural closure is mandatory to avoid GSF recurrence or CSF leakage occurrence [14].

Usually, dural tear extends beyond the fracture borders, that is way the fracture should be extended to craniectomy, until normal dura is reveled [15,27,30,31]. In many cases, dural tears may involve transverse or sagittal sinus [23]. In such cases bleeding from the dural sinus can occur, that is life threatening in these age groups. Even if the sinus is not torn, its vicinity may pose problems regarding dural repair. If the fracture is parallel to the sinus, dural repair is difficult, because of the narrowness of the dural edge to the venous sinus. If proximity of the sinus does not allow graft suture with borders of normal dura mater, the graft can be sutured with dura across the sinus after extending the craniectomy or directly to the skull's edge above the sinus. If the fracture is perpendicularly to the sinus, the end closest to the venous sinus does not need repairing. We extended the fracture to a right frontoparietal craniectomy over the coronal suture, reaching up to the anterior fontanelle and suturing a patch of periosteum to free dural borders. 

We do not recommend cranioplasty in children under 3 years old, because a child's head is growing, and there is a high risk of bone graft displacement [14]. Plus, new bone apposition within the skull defect occurs in most children and in infants dura mater has osteogenic potential [32–35]. Cranioplasty with methylmethacrylate was reported to have good results on long–term follow–up [13]. 

CSF shunt diversion is indicated in CSF leakage in patients with adequate treatment of GSF and concomitant hydrocephalus

Finding the group of patients carrying risk of developing GSF immediately after trauma is challenging. To develop a GSF, the diastatic fracture and dural tear are mandatory. The age 0–3 years old is a predisposed factor for GSF. Cranial X–rays and cerebral CT–scan cannot show dural tears [12,18,22,23]. Cerebral MRI can indent dural tears, and has a high predictive value of developing GSF, but, unfortunately, we, like many other neurosurgical departments, do not have an MRI in emergency. Ultrasonography through diastatic fracture line can identify dural tears [12,19].

#### Skull base fractures

Patients with skull base fractures present some characteristic signs, such as periobitar (‘raccoon's eyes’) or postauricular ecchymosis (Battle's sign), epistaxis, otorhagia, etc. The diagnostic of skull base fracture is made on CT–scan. The consequence of skull fractures is CSF leakage, with rhinoliquorea or otoliquorea. In young children, 0–5 years old frontobasal fractures are common [36]. 

Surgery is required in persistent CSF leakage, with lack of response to conservative therapy (lumbar punction, or lumbar drainage). Closing the dural tear, and if possible bone defect is the key step in surgery. Several approaches can be achieved: classical, transcranial extra– or intradural, or transnasal transsfenoidal endoscopic in CSF leakage with rhinoliquorea, difficult in children. Defects within the cribriform plate, planum sphenoidale, superomedial surface of the orbit, and the posterior surface of the petrous bone, may be closed through either intradural or extradural approaches. Intradural approaches are fit for defects into the sphenoidal wing, or tip of the petrous bone [37]. Indirect transnasal transsfenoidal endoscopic approach consists of packing the sphenoidal sinus with fat tissue, muscle, and a bone [37]. In many cases, a multidisciplinary team (neurosurgeon, ophthalmologist, oromaxilofacial surgeon) is needed for dural and craniofacial skeleton repairing [36].

#### Penetrating head injury (PHI)/Craniocerebral wounds in children

PHI are rare in children [38]. In PHI, all anatomic structures, scalp, skull, dura mater, leptomeningeal layer and brain parenchyma are involved. Many bizarre injuries 

Clinical features may be:

- brief posttraumatic LOC, followed by lucid interval for several hours, and then progressive alteration of the level of consciousness
- persistent posttraumatic coma
- posttraumatic coma, followed by the regaining of consciousness
- posttraumatic conservation of consciousness without coma
- posttraumatic conservation of consciousness with delayed coma

Clinics in newborns and infants are vague consisting of hypothonia, seizures, and tense fontanelle. 

EDH in children with hydrocephalus and ventricular shunt is a serious and urgent chapter of children pathology. In such children, CT is mandatory immediately. Even after a mild TBI usually initially asymptomatic, coma may rapidly onset. CT–scan must be performed without delay [39]. Usually, there is no lucid period, rapid alteration of level of consciousness immediately after trauma and severe impairment of vegetative functions. The supratentorial EDH patients present with motor deficits, jacksonian seizures, anisocoria, and comatose state [40]. 

Posterior fossa EDH occurs less frequently than supratentorial ones, but it is the most common posttraumatic space–occupying lesion of the posterior fossa in children [41]. Posterior fossa EDH blocks cistern magna, causing brainstem compression and obstructive hydrocephalus with acute intracranial hypertension. Children with EDH posterior fossa may have a rapid deterioration without significant warning symptoms and may result in death.

The positive diagnosis, location and size of EDH are made on CT–scan. EDHs are extraaxial lenticular–shaped masses situated between the dura and the inner table of the skull, with density varying according to the age of the lesion. A normal CT–scan immediately after trauma, does not exclude the possibility of further development of an EDH.

Children with skull fractures, presenting with vomiting or alteration of the level of consciousness, must be clinically observed together with a CT–scan for the development of an EDH. 

Surgery is indicated according to the clinical status and CT–scan:

- Comatose patient with anisocoria, and CT–scan showing EDH require urgent surgery
- Coma and worsening of neurological state in case of EDH's volume > 25 ml
- EDH's volume > 30 ml, even in the absence of clinical signs
- EDH's volume > 25 ml, if EDH is located within the posterior fossa or temporal region
- Midline shift > 4 mm, with worsening of neurological status
- EDH volume increase

Surgery consists of the hematoma evacuation. One of the most important therapeutic targets is blood replacement in the shocked child, which should be done within 20–30 minutes, before starting the operation

Conservative treatment can be attempted in an alert child, with no focal neurological deficits, in which CT–scan showed an EDH having a volume < 25 ml, with a thickness <10 mm and midline shift < 4 mm. The child must be held in a neurosurgical center, where surgery can be performed if needed, under attentive clinical observation and  CT–scan must be repeated [42]. 

#### Diffuse brain swelling (DBS)

Diffuse brain swelling in newborns is due to birth asphyxia and secondary reperfusion. DBS is mixed by vasogenic and cytotoxic edema

The diagnosis is established by CT–scan and the treatment is specific, according to the clinical status. The particularity of DBS in children is represented by its early onset. It is very severe and it is often followed by diffuse cerebral ischemia (‘black brain’).

Posttraumatic DBS can accompany any cerebral lesion in children and it requires intracranial pressure (ICP) monitoring. After failure of conservative therapy, bilateral decompressive craniectomy may be needed as the last resort treatment strategy. In children, decompressive craniectomy improves the neurological outcome and diminishes death risk [43,44]. The necessity of decompressive craniectomy and duraplasty is especially important in children, due to the lack of response to usual therapies, in the treatment of intracranial hypertension [45,46]. 

The surgical technique for diffuse brain swelling: wide bilateral hemicraniectomy, dural opening in a stellate fashion, dural graft to increase the available volume before closure, and finally wound closure. 

## Conclusions

Neurotrauma pathology is very different in infants and toddlers compared to different age patients. Accurate and rapid clinical and neuroimagistic diagnosis is the key of success. The Pediatric Neurosurgical Department and Pediatric Intensive Care Unit represent a vital necessity. Long time follow–up is mandatory.  

CT–scan is the main investigation tool and must be performed in all children with TBI, in the first three hours. 

Hemorrhagic shock may rapidly occur in infants and young children. 

Grow skull fracture is a specific posttraumatic lesion in infants and young children. Surgery is always required to prevent neurological deficits and/or seizures occurrence.

Tight dural closure is the key step in surgical management. Duraplasty can be done with a patch of pericranium / periosteum. Cranioplasty is not indicated in infants.
